# Genetic Variation Between Small Bowel and Colon-Predominant Crohn's Disease

**DOI:** 10.1016/j.jcmgh.2024.02.010

**Published:** 2024-02-17

**Authors:** Halee Patel, R. Alan Harris, Justin H. Qian, Numan Oezguen, Ashleigh Watson, Reka G. Szigeti, Stanley Cho, Wenly Ruan, Savini Britto, Antone Opekun, Geoffrey Preidis, Richard Kellermayer

**Affiliations:** Division of Gastroenterology, Hepatology and Nutrition, Department of Pediatrics, Baylor College of Medicine, Texas Children’s Hospital, Houston, Texas; Human Genome Sequencing Center, Department of Molecular and Human Genetics, Baylor College of Medicine, Houston, Texas; Division of Gastroenterology, Hepatology and Nutrition, Department of Pediatrics, Baylor College of Medicine, Texas Children’s Hospital, Houston, Texas; Department of Pathology and Immunology, Baylor College of Medicine, Houston, Texas; Texas Children's Microbiome Center, Department of Pathology, Texas Children's Hospital, Houston, Texas; Division of Gastroenterology, Hepatology and Nutrition, Department of Pediatrics, Baylor College of Medicine, Texas Children’s Hospital, Houston, Texas; Clinical Pathology and Genomic Medicine, Methodist Hospital, Weill Cornell Medical College, Houston, Texas; Division of Gastroenterology, Hepatology and Nutrition, Department of Pediatrics, Baylor College of Medicine, Texas Children’s Hospital, Houston, Texas; Department of Gastroenterology, Baylor College of Medicine, Texas Children’s Hospital, Houston, Texas; Division of Gastroenterology, Hepatology and Nutrition, Department of Pediatrics, Baylor College of Medicine, Texas Children’s Hospital, Houston, Texas; Division of Gastroenterology, Hepatology and Nutrition, Department of Pediatrics, Baylor College of Medicine, Texas Children’s Hospital, Houston, Texas; United States Department of Agriculture Children’s Nutrition and Research Center, Houston, Texas

The genomic contribution to inflammatory bowel diseases (IBDs) is complex and polygenic,^1^ with more than 240 susceptibility loci identified by genome-wide association studies.^2^ Although genome-wide association studies are powerful, those still carry limitations, and commonly are not powered toward rare subtype analysis within a disease group.^3^ Therefore, in this study, we set out to compare the genetics (exomes) of patients with proximal small-bowel–predominant Crohn’s disease (SB-CD) (L4) or colon-predominant Crohn’s disease (C-CD) (L2 and/or colon-predominant L3). We also examined a differentiating candidate gene in a mouse model of colitis to address the translational relevance of our findings. Susceptibility subject–gene bipartite networks in SB-CD and C-CD also were generated to study the polygenic background of the disease subtypes.

Eight SB-CD and 11 C-CD cases met inclusion criteria. The clinical characteristics of these patients are included in Table 1. With methods described in the Supplementary Materials, we identified 115 single-nucleotide polymorphisms (SNPs) with a combined annotation-dependent depletion (CADD) (a tool for scoring the deleteriousness of single-nucleotide variants) Phred score >10 associated with 97 genes, which had significantly (*P* < .01) different allele variation between C-CD and SB-CD. An SNP in the *EFNA3* gene was among the top 28 candidates with a CADD score >20 to differentiate between the 2 phenotypically distinct CD groups (Supplementary Table 1). *EFNA3* rs17723260 (predicted to be deleterious) was found to have a significantly lower allele frequency (4.5%) in C-CD, compared with its allele frequency of 37.5% in SB-CD (chi square *P* = .0097). This finding indicated that *EFNA3* might play a role in modulating colonic inflammation, in which a deleterious genetic defect might provide protection against colitis (and direct autoimmunity against the proximal small bowel) in the polygenic background of CD. Importantly, *EFNA3* has been linked to ulcerative colitis^4^ and CD^5^ as well. The other 4 genes associated with the top 5 SNP candidates (Supplementary Table 1) already have been connected with IBD or mammalian intestinal inflammation. *ACACB* up-regulated in small-bowel strictures of the marmoset,^6^ *STEAP1B* linked to adult IBD,^7^ *DSG1*as a serologic marker of complicated CD,^8^ and *CYP4F2* in which the specific polymorphism identified in this study (rs2108622) also has been observed to associate significantly with CD by Costea et al.^9^

**Table 1:** Demographic and Baseline Characteristics of Patients With Either C-CD or SB-CD

	C-CD (n = 11)	SB-CD (n = 8)	*P* value
Mean age at diagnosis, *y*	10.9	11	.95
Sex, % female	36.3	25	1
Ethnicity, % Caucasian	72.7	87.5	.6
Paris location, *%*			
L1	0	50	.018
L2	54.5	0	.018
L3	45.5	25	.63
L4a	18.2	37.5	.6
L4b	0	100	.0001
Paris behavior, *%*			
B1	45.5	12.5	.177
B2	27.2	62.5	.18
B3	18.2	0	.485
B2/B3	9.1	25	.546
Perianal disease, % yes	36.4	12.5	.338
Presence of granulomas, % yes	33.3 (2/6)	33.3 (3/9)	1
Surgical intervention required, % yes	54.5	75	.633
Surgery in first 2 years, % yes	33.3 (2/6)	50 (3/6)	1
Type of surgery, *%*	n = 6	n = 6	
Partial colectomy	50	50	1
Total colectomy	33.3	0	.455
Ileocecotomy	0	16.7	1
Enterectomy	16.7	66.7	.242
Small-bowel diversion only	16.7	0	1
Biologic agents used before surgery, *%*	n = 6	n = 6	
Infliximab	100	66.7	.455
Adalimumab	83.3	33.3	.242
Ustekinumab	16.7	16.7	1
Vedolizumab	16.7	0	1
Biologic agents used in nonsurgical patients, *%*	n = 5	n = 2	
Infliximab	80	100	.49
Adalimumab	60	0	.429

C-CD, colon-predominant Crohn’s disease; SB-CD, small-bowel–predominant Crohn’s disease.

A dextran sodium sulfate (DSS) model experiment with 5 wild-type and 5 *Efna3* null-allele female mice found *Efna3* null mice to be protected significantly (*P* < .0001) against colitis (Supplementary Figure 1). We validated these findings in a subsequent experiment (Supplementary Figure 2). Male *Efna3* null-allele mice did not demonstrate a significant difference in DSS colitis severity compared with wild-type littermates (not shown). Based on the literature and our murine model findings, we propose that normal or increased *EFNA3* expression in patients with CD might shift susceptibility toward complicated colonic and terminal ileal (ie, L1, L2, and L3) disease. On the contrary, decreased *EFNA3* expression might shift the disease toward the more proximal L4b phenotype by protecting against colonic injury in the background of CD.

**Figure 1:**
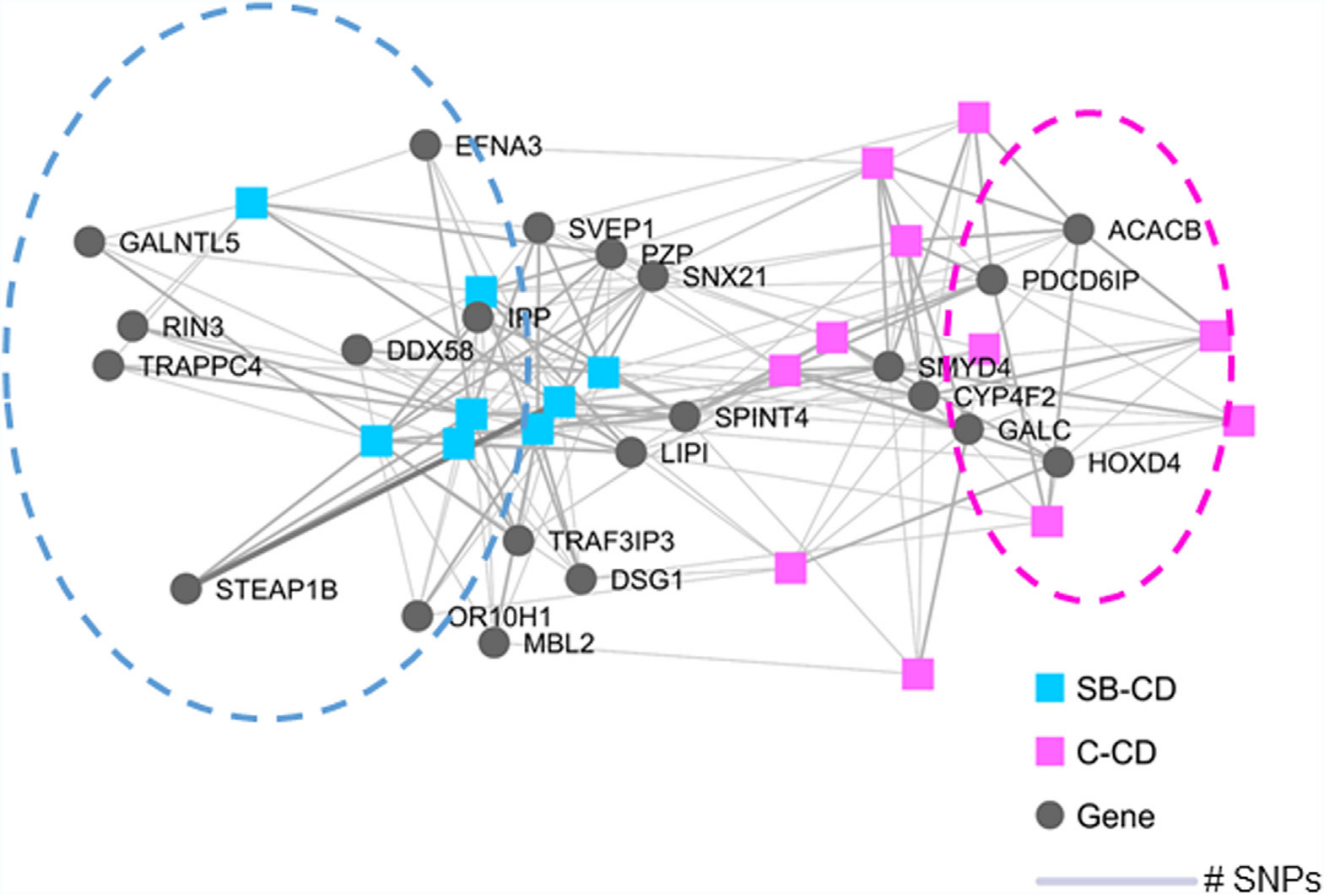
**Bipartite subject–gene networks between combined annotation-dependent depletion (CADD) >20, missense single-nucleotide polymorphism (SNP)-associated genes that differentiated between small-bowel–predominant Crohn’s disease (SB-CD) and colon-predominant Crohn’s disease (C-CD).** The nodes represent either a subject or a gene and the edges represent the presence of polymorphism(s). The thickness of the edges is proportional to the number of SNPs in the corresponding subject–gene pair. Differential cluster of genes separated SB-CD (highlighted by *blue circle*) from C-CD (highlighted by *pink circle*).

Recognizing the polygenic nature of most IBD cases, we studied the disease subtype differentiating subject–gene networks within our patients considering genes that had CADD >20 prediction for deleteriousness (ie, high susceptibility genes) (Supplementary Table 1). Differential gene networks separated SB-CD from C-CD (Figure 1), in which *EFNA* was associated with SB-CD, among others. Although there was no clear clustering of the differentiating genes in SB-CD when analyzed separately (Supplementary Figure 3*A*), we observed a commonly shared network between *ACACB*, *CYP4F2*, *GALC*, *HOXD4*, *PDCD6IP*, *SPINT4*, and *SMYD4* in C-CD patients (Supplementary Figure 3*B*).

This was a genetic study to address exome (coding) variation between C-CD (L2 or colon-predominant L3) and SB-CD (L4b). We describe a candidate gene compendium (Supplementary Table 1) in which SNPs predicted to be deleterious varied significantly in abundance between the 2 patient groups. Although our cohort sizes were small, they were sufficient to yield significant results, indicating that genetic predisposition may direct intestinal disease location in the background of pediatric CD. As a comparison, identical methodology (PLINK) (see Supplementary Materials) examining exome variation between granulomatous and nongranulomatous CD^10^ did not find significant separation, although the cohorts in that study were larger than within this work. The existing literature supports the significance of our results (Supplementary Materials). Even beyond the top candidates, there are numerous other genes within our compendium that have been implicated in IBD.

In summary, the biomedical literature and our mouse model findings implicate the translational relevance of our candidate gene compendium for directing colon- vs small-bowel–predominant CD development. We trust that our findings will be replicated in larger CD cohorts differentiated by disease location. Our work may set the nidus for CD subtype–based precision medicine by guiding individualized treatment strategies.
