# Supplementary material for: Genetic Variation Between Small Bowel and Colon-Predominant Crohn's Disease
Source: Cell Mol Gastroenterol Hepatol. 2024 Feb 17;17(6):1069–71. doi: 10.1016/j.jcmgh.2024.02.010 (PMC11127623; doi:10.1016/j.jcmgh.2024.02.010)
Supplement: Supplementary Materials [file mmc1.pdf]

Supplementary Materials to "Genetic Variation between Small Bowel and Colon Predominant Crohn Disease"

dSNP ID	Gene Symbol	Plink p value (Chi-square)	C-CD Frequency (%)	SB-CD Frequency (%)	C-CD/SB-CD abundance	CADD PHRED Score
**rs17723260**	*EFNA3*	0.009673	4.545	37.5	0.1212	25.8
**rs2300455**	*ACACB*	0.001946	54.55	6.25	8.728	25.6
**rs16881812**	*STEAP1B*	0.004898	0	31.25	0	25.6
**rs3752095**	*DSG1*	0.004691	9.091	50	0.18182	25.4
**rs2108622**	*CYP4F2*	0.002586	68.18	18.75	3.636267	24.8
**rs149580813**	*STEAP1B*	0.004898	0	31.25	0	24.8
**rs117068593**	*RIN3*	0.004898	0	31.25	0	24.5
**rs6960270**	*GALNTL5*	0.004898	0	31.25	0	24.4
**rs398607**	*GALC*	0.007982	54.55	12.5	4.364	24.3
**rs6017667**	*SPINT4*	0.008576	31.82	75	0.424267	23.8
**rs1889323**	*SVEP1*	0.005268	13.64	56.25	0.242489	23.8
**rs1800450**	*MBL2*	0.009673	4.545	37.5	0.1212	23.7
**rs2822432**	*LIPI*	0.000461	18.18	75	0.2424	23.3
**rs28375469**	*IPP*	0.004691	9.091	50	0.18182	23
**rs11549830**	*SMYD4*	0.008576	31.82	75	0.424267	22.9
**rs3203777**	*PDCD6IP*	0.003719	59.09	12.5	4.7272	22.5
**rs3213831**	*PZP*	0.00517	18.18	62.5	0.29088	22.4
**rs1566452**	*WWP2*	0.004691	9.091	50	0.18182	22.1
**rs3802881**	*TRAPPC4*	0.004898	0	31.25	0	21.9
**rs1051861**	*ARID4A*	0.003623	27.27	75	0.3636	21.7
**rs4808383**	*OR10H1*	0.009673	4.545	37.5	0.1212	21.6
**rs669694**	*TRAF3IP3*	0.004691	9.091	50	0.18182	21.5
**rs10414643**	*PRR12*	0.008513	45.45	6.25	7.272	21
**rs4638862**	*SNX21*	0.00517	18.18	62.5	0.29088	20.9
**rs17473148**	*COPS2*	0.003405	40.91	0	#DIV/0!	20.7
**rs34727427**	*HOXD4*	0.008513	45.45	6.25	7.272	20.6
**rs1250**	*GPBP1L1*	0.004691	9.091	50	0.18182	20.3
**rs10813831**	*DDX58*	0.009673	4.545	37.5	0.1212	20.1

Supplementary Table 1: The top gene candidates (according to CADD scoring of >20) associated SNPs that differentiated between colon predominant (C-CD) and small bowel predominant (SB-CD) Crohn's disease.

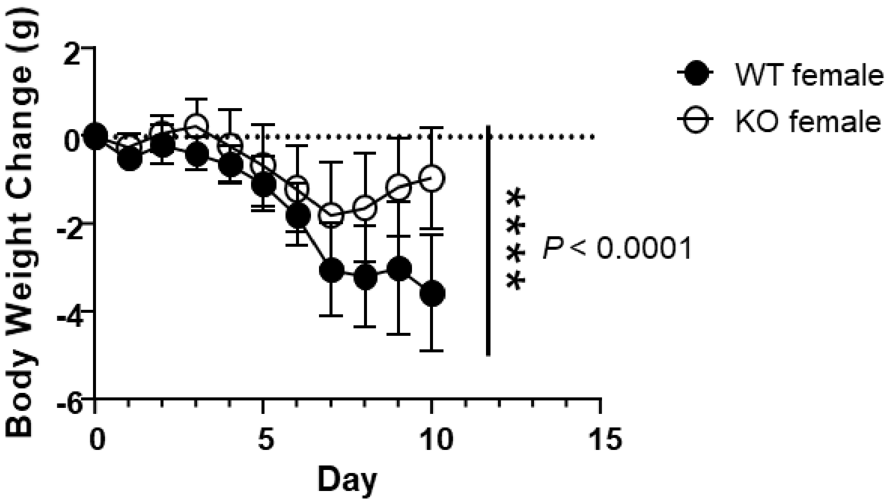

Supplementary Figure 1. Weight loss curves during the dextran sodium sulfate (DSS) experiment. Weight loss, a reliable single outcome measure of DSS colitis severity, was significantly less in in the discovery cohort of *Efna3* null-allele (KO) mice compared to litter mate wild-type (WT) controls. DSS exposure in the drinking water lasted for the first 5 days of the experiment. N=5 in both groups

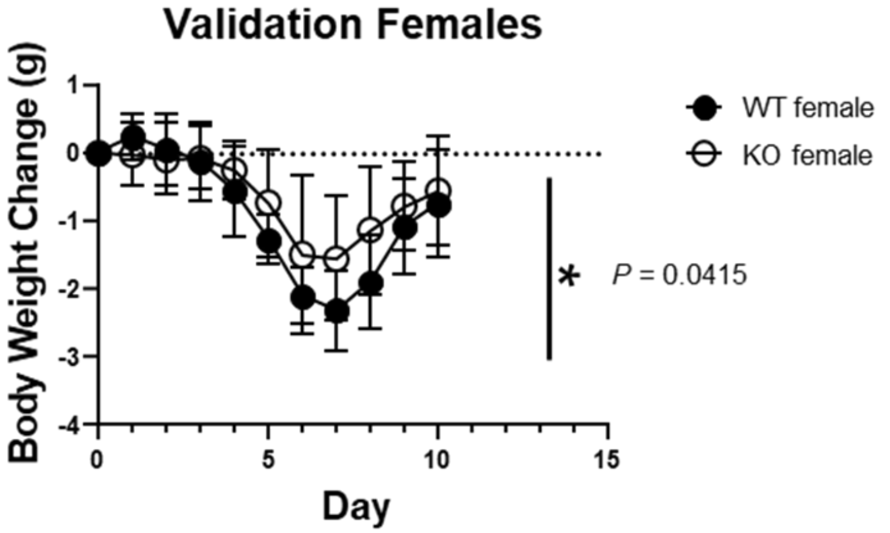

Supplementary Figure 2. Weight loss curves during the validation dextran sodium sulfate (DSS) experiment. Weight loss, a reliable single outcome measure of DSS colitis severity, was significantly less in in the discovery cohort of *Efna3* null-allele (KO) mice compared to litter mate wild-type (WT) controls. DSS exposure in the drinking water lasted for the first 5 days of the experiment. WT n= 6, *Efna3* KO n=8.

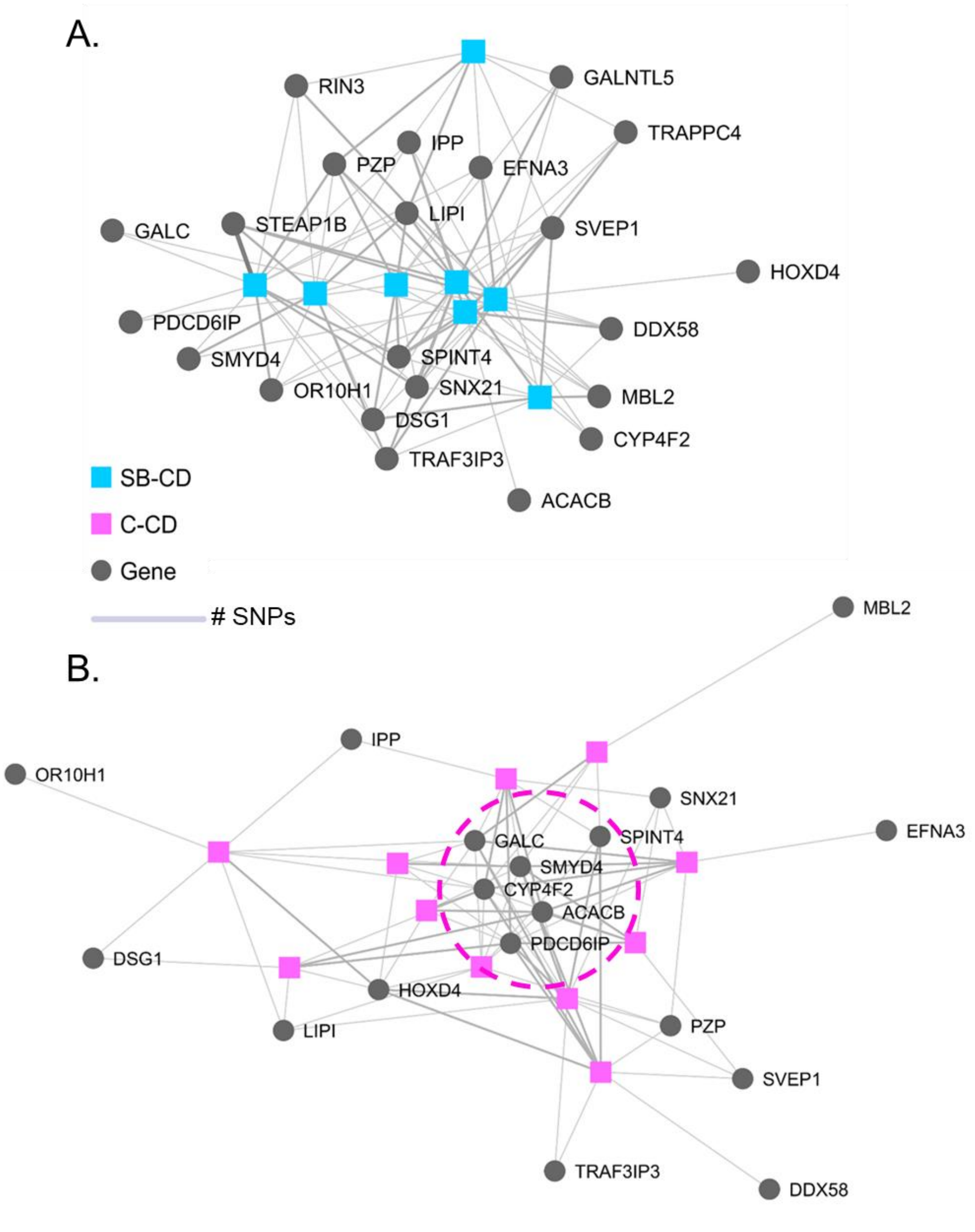

Supplementary Figure 3. **A.** Separate analysis of the susceptibility gene networks in SB-CD. No clear clustering of shared genes was observed. **B.** Separate analysis of the susceptibility gene networks in C-CD. The most commonly shared susceptibility genes (*ACACB, CYP4F2, GALC, HOXD4, PDCD6IP, SPINT4, and SMYD4*) clustered in the center (dotted circle) of the network.

Supplemental Discussion:

Sexual dimorphism has been demonstrated in murine models of colitis linking to estrogens, and females may serve as more sensitive representatives in some translational studies.[1]

The *EFNA3* gene encodes the protein Ephrin A3 (EFNA3), which is a member of the ephrin protein family.[2] Ephrins are ligands for the Eph (erythropoietin-producing hepatocellular carcinoma) receptors. Increased expression of *EFNA3* along with other genes in the Eph-ephrin family such as *EFNB2, EPHA10* and *EPHA1* was found in UC patients in remission.[3] Bakirtzi *et al.*[4] found that neurotensin promotes TNBS colitis and angiogenesis in mice through the micro-RNA 210 (miR-210) - *Efna3* pathway. Ephrins and their receptors have been proposed as therapeutic targets in IBD.[5] *EFNB2* (Ephrin-B2) was found to be up-regulated in both perilesional and lesional intestinal epithelial cells of CD patients.[6] Another recent publication connected *EFNA3* with CD.[7] The patients in this study had either isolated terminal ileal (L1) or ileocecal/ileocolonic (L3) disease. While this study did not detect differentially variable positions (DVPs, or differentiating SNPs), it is intriguing that our candidate SB-CD predominant *EFNA3* SNP (chr1:155,086,187) is only 1,211 bases downstream from the *EFNA3* DMP of Ventham NT, *et al.*[7] indicating this intron region to likely be important in transcription regulation.

Our network analyses showed that there might be a more commonly shared genetic network in C-CD patients as opposed to SB-CD, which could direct their phenotypic separation. This shared network in C-CD included *ACACB, CYP4F2, GALC, HOXD4, PDCD6IP, SPINT4,* and *SMYD4*. The observation of *ACACB* being upregulated in small bowel strictures of the marmoset,[8] and our finding indicates its dosage based influence in the background of CD. Opposite of what we proposed for *EFNA3*, increased expression of *ACACB* might contribute to stricturing complications of SB-CD, while deficiency of the gene may shift the disease towards

colitis. As already discussed, *CYP4F2* has been linked to CD in pediatric patients.[9] *GALC* has been recognized among genes relevant for glycosylation in respect to homeostasis and gut microbiota interactions in IBD.[10] Differential methylation of *HOXD4* has been recently demonstrated in the rectal mucosa of patients with ulcerative colitis.[11] Increased mucosal expression of *PDCD6IP* was observed in UC patients who experienced long-term remission after discontinuation of adalimumab,[12] indicating a protective role of this gene against colitis in the background of IBD.

Material and Methods:

Patients

Pediatric patients with CD gave informed consent and were enrolled into the PRO-KIIDS PILOT STUDY TO EXAMINE SPECIFIC GENOTYPES AND PROTEOTYPES THAT INCREASE THE RISK FOR COMPLICATED CROHN'S DISEASE (H-43617).

Exome sequencing

Whole-exome next-generation sequencing (WES) was performed from peripheral blood-derived DNA. After DNA quality control tests, Illumina sequencing libraries with incorporated barcodes were produced following standard procedures.[13] Bar-coded samples were pooled and captured together. The resulting pools, enriched for the human exome by the capture process, were sequenced using the Illumina NovaSeq instrument.

SNP calling and annotation

The Illumina 150bp paired-end reads were aligned to the human GRCh38 reference genome assembly using BWA MEM with an average on-target sequence depth of 59.1X across the samples. Picard MarkDuplicates version 1.105 (http://broadinstitute.github.io/picard/) was used to identify and mark duplicate reads. The GATK v 4.1.2.0 best practices pipeline[14,15] was used to identify single nucleotide polymorphisms (SNPs). Variant Effect Predictor software (VEP)[16] v98 was used to annotate variants based on merged Ensembl and RefSeq gene models. The potential deleteriousness of SNPs was determined by the Combined Annotation Dependent Depletion (CADD) v1.5 scoring tool.[17]

Exome Data analysis

PLINK[18] was used to identify SNPs that were overrepresented in comparisons between the groups based on chi-square allelic tests. Due to the small, but highly selective sample sizes, the level of significance for exome wide differentiation was relaxed to $p<0.01$ for the PLINK-based group comparison.

Animal Studies

Eight-week-old C57BL/6-$Efna3^{tm1Ebp}$/J mice were obtained from The Jackson Laboratory (Bar Harbor, ME, USA) and maintained in accordance with the National Institutes of Health (NIH) Guide for the Care and Use of Laboratory Animals (IACUC protocol AN-5351). The filial generation 0 (F0) of the mice were mated and two subsequent generations (F1 and F2) of the *Wild-type (WT)* and *Efna3* null-allele mice were bred. Each generation was mated at 10-12

weeks of age to produce the subsequent generation. Experiments were performed in the IACUC-approved CNRF facility of the Children's Nutrition Research Center.

When the F2 mice reached 16-21 weeks of age, designated as "adult" mice, the WT and *Efna3* null-allele animals were cohoused separated by gender, but as littermates, for a period of 10-14 days. All mice (while still cohoused) were exposed to 3% DSS (MW = 40,000 – 50,000, VWR SUMMUS Industries, Sugar Land, TX, USA) in the drinking water for 5 days or when the average weight loss in either group reached greater than 5%. Starting from initiation of DSS, daily body weights were measured for 10 days as the single outcome measure of DSS-induced colitis severity.[1] We have previously highlighted that weight loss alone is a sufficient, practical, and cost-effective measure of colitis severity in DSS murine model.[19] Subsequently, mice were euthanized per the IACUC-approved protocol for isoflurane overdosing.

Network analyses

The information about high susceptibility (CADD>20, missense to increase functional relevance potential) SNPs (i.e. minor alleles, see Supplementary Table 1) in subjects were visualized in form of bipartite subject-gene networks. In these networks, the nodes represent either a subject or a gene (in which a SB-CD vs C-CD differentiating susceptibility SNP was identified) and the edges represent the presence of the polymorhism(s). The thickness of the edges is proportional to the number of SNPs in the corresponding subject-gene pair. The networks were created utilizing the Edge-weighted Spring Embedded layout algorithm within Cytoscape[20] version 3.10.0.

Statistical Analysis of Weight Changes

Analysis of variance (ANOVA) with repeated measures was performed using R to evaluate animal body weights and percent change in body weights across the experiment. A paired t-test was used to analyze the difference in colonic lengths. P-values less than 0.05 were considered significant.

Supplementary References

1. Krishna M, Engevik M, Queliza K*, et al.* Maternal lactobacillus reuteri supplementation shifts the intestinal microbiome in mice and provides protection from experimental colitis in female offspring. *FASEB Bioadv* 2022;**4**:109-20.
2. Darling TK, Lamb TJ. Emerging roles for eph receptors and ephrin ligands in immunity. *Front Immunol* 2019;**10**:1473.
3. Fenton CG, Taman H, Florholmen J, Sorbye SW, Paulssen RH. Transcriptional signatures that define ulcerative colitis in remission. *Inflamm Bowel Dis* 2021;**27**:94-105.
4. Bakirtzi K, Law IK, Xue X*, et al.* Neurotensin promotes the development of colitis and intestinal angiogenesis via hif-1alpha-mir-210 signaling. *J Immunol* 2016;**196**:4311-21.
5. Grandi A, Zini I, Palese S*, et al.* Targeting the eph/ephrin system as anti-inflammatory strategy in ibd. *Front Pharmacol* 2019;**10**:691.
6. Hafner C, Meyer S, Langmann T*, et al.* Ephrin-b2 is differentially expressed in the intestinal epithelium in crohn's disease and contributes to accelerated epithelial wound healing in vitro. *World J Gastroenterol* 2005;**11**:4024-31.
7. Ventham NT, Kennedy NA, Kalla R*, et al.* Genome-wide methylation profiling in 229 patients with crohn's disease requiring intestinal resection: Epigenetic analysis of the trial of prevention of post-operative crohn's disease (toppic). *Cell Mol Gastroenterol Hepatol* 2023;**16**:431-50.
8. Sheh A, Artim SC, Burns MA*, et al.* Common marmoset gut microbiome profiles in health and intestinal disease. *bioRxiv* 2020:2020.08.27.268524.
9. Costea I, Mack DR, Israel D*, et al.* Genes involved in the metabolism of poly-unsaturated fatty-acids (pufa) and risk for crohn's disease in children & young adults. *PLoS One* 2010;**5**:e15672.
10. Kudelka MR, Stowell SR, Cummings RD, Neish AS. Intestinal epithelial glycosylation in homeostasis and gut microbiota interactions in ibd. *Nat Rev Gastroenterol Hepatol* 2020;**17**:597-617.
11. Cheng B, Rong AM, Li W, Bi X, Qiu X. Dnmt3a-mediated enterocyte barrier dysfunction contributes to ulcerative colitis via facilitating the interaction of enterocytes and b cells. *Mediators Inflamm* 2022;**2022**:4862763.
12. Sakurai T, Nishiyama H, Sakai K*, et al.* Mucosal microbiota and gene expression are associated with long-term remission after discontinuation of adalimumab in ulcerative colitis. *Sci Rep* 2020;**10**:19186.
13. Lupski JR, Gonzaga-Jauregui C, Yang Y*, et al.* Exome sequencing resolves apparent incidental findings and reveals further complexity of sh3tc2 variant alleles causing charcot-marie-tooth neuropathy. *Genome Med* 2013;**5**:57.

14. McKenna A, Hanna M, Banks E*, et al.* The genome analysis toolkit: A mapreduce framework for analyzing next-generation DNA sequencing data. *Genome Res* 2010;**20**:1297-303.
15. DePristo MA, Banks E, Poplin R*, et al.* A framework for variation discovery and genotyping using next-generation DNA sequencing data. *Nat Genet* 2011;**43**:491-8.
16. McLaren W, Gil L, Hunt SE*, et al.* The ensembl variant effect predictor. *Genome Biol* 2016;**17**:122.
17. Rentzsch P, Witten D, Cooper GM, Shendure J, Kircher M. Cadd: Predicting the deleteriousness of variants throughout the human genome. *Nucleic Acids Res* 2019;**47**:D886-D94.
18. Purcell S, Neale B, Todd-Brown K*, et al.* Plink: A tool set for whole-genome association and population-based linkage analyses. *Am J Hum Genet* 2007;**81**:559-75.
19. Britto SL, Krishna M, Kellermayer R. Weight loss is a sufficient and economical single outcome measure of murine dextran sulfate sodium colitis. *FASEB Bioadv* 2019;**1**:493-7.
20. Shannon P, Markiel A, Ozier O*, et al.* Cytoscape: A software environment for integrated models of biomolecular interaction networks. *Genome Res* 2003;**13**:2498-504.
